# The impact of a standardized Enhanced Recovery After Surgery (ERAS) protocol in patients undergoing minimally invasive heart valve surgery

**DOI:** 10.1371/journal.pone.0283652

**Published:** 2023-03-24

**Authors:** Alexander Gebauer, Johanna Konertz, Johannes Petersen, Jens Brickwedel, Denise Köster, Leonie Schulte-Uentrop, Hermann Reichenspurner, Evaldas Girdauskas

**Affiliations:** 1 Department of Cardiovascular Surgery, University Heart & Vascular Center Hamburg, Hamburg, Germany; 2 Institute of Medical Biometry and Epidemiology, University Medical Center Hamburg-Eppendorf, Hamburg, Germany; 3 Department of Anesthesiology, Center of Anesthesiology and Intensive Care Medicine, University Medical Center Hamburg-Eppendorf, Hamburg, Germany; 4 Department of Cardiothoracic Surgery, University Medical Center Augsburg, Augsburg, Germany; Ataturk University Faculty of Medicine, TURKEY

## Abstract

**Background:**

An enhanced recovery after surgery (ERAS) protocol is a multimodal and multi-professional strategy aiming to accelerate postoperative convalescence. Pre-, intra- and postoperative measures might furthermore reduce postoperative complications and hospital length of stay (LOS) in a cost-effective way. We hypothesized that our unique ERAS protocol leads to shorter stays on the intensive care unit (ICU) and a quicker discharge without compromising patient safety.

**Methods:**

This retrospective single center cohort study compares data of n = 101 patients undergoing minimally invasive heart valve surgery receiving a comprehensive ERAS protocol and n = 111 patients receiving routine care. Hierarchically ordered primary endpoints are postoperative hospital length of stay (LOS), postoperative complications and ICU LOS.

**Results:**

Patients risk profiles and disease characteristics were comparably similar. Age was relevantly different between the groups (56 (17) vs. 57.5 (13) years, p = 0.015) and therefore adjusted. Postoperative LOS was significantly lower in ERAS group (6 (2) days vs. 7 (1) days, p<0.01). No significant differences, neither in intra- or postoperative complications, nor in the number of readmissions (15.8% vs. 9.9%, p = 0.196) were shown. In hospital LOS (7 (3) days vs. 8 (4) days, p<0.01) and ICU LOS (18.5 (6) hours vs. 26.5 (29) hours, p<0.01) a considerable difference was shown.

**Conclusion:**

The ERAS protocol for minimally invasive heart valve surgery is safe and feasible in an elective setting and leads to a quicker hospital discharge without compromising patient safety. However, further investigation in a randomized setting is needed.

## Introduction

Enhanced recovery after surgery (ERAS) protocols are multimodal and multi professional strategies in perioperative care, aiming to reduce hospital length of stay (LOS) and healthcare-associated complications by attenuating physiological and psychological stress responses [1–3].

ERAS was initially established in 1990 by danish surgeon Prof. Dr. H. Kehlet and was based on the assumption, that trauma-induced stress responses stimulate endocrine and metabolic changes, which play a crucial role in the appearance of postoperative morbidity and prolonged hospital stays [3]. Early key elements included the utilization of minimal invasive surgery to reduce trauma, early mobilization after surgery and a new nutritional intake regimen [3]. With growing insights into the development of multilayered stress responses, these protocols evolved to cover up the entire patient journey, starting from preoperative patient education up to the point of early ambulation and follow-up care [4]. This is why a successful implementation requires the interdisciplinary cooperation of surgeons, anesthetists, physiotherapists and nurses [5]. Thus, physiologic and psychologic stress responses are supposed to be reduced, resulting in an overall enhancement of recovery [6].

ERAS protocols proved to be efficient most notably in colorectal surgery [7, 8], demonstrating major advantages in healthcare-associated infections, hospital LOS, gastrointestinal morbidity [9, 10], and cost-effectiveness [11]. Subsequently, an employment in different surgical settings was suggested [12]. These implications led to a paradigm shift in perioperative care, as ERAS Society was established in 2010 and since then developed certified recommendations for numerous surgical disciplines.

For the matter of cardiac surgery (CS), patients offer a wide variety of complex pathologies and cardiac disease typically coexists with multiple comorbidities. In addition, the use of cardiopulmonary bypass (CPB) can trigger systemic inflammatory response syndromes (SIRS) [13], which adds to the challenge of designing a protocol that addresses the effects on multiple organ systems and their impact on the postoperative recovery phase [14].

This is why ERAS Cardiac Society, a subgroup within ERAS Society, developed evidence based expert consensus recommendations in the spirit of basic ERAS principles through a systematic literature review process. This scalable guideline manuscript provides a summarization of key elements for deployment in CS, marked with class of evidence and level of recommendation [15]. Nevertheless, the majority of protocols in CS suggests feasibility of applying certain elements of ERAS [16, 17], but hardly any cover up the entire clinical process provided by ERAS and high quality data is missing.

The program designed at the University Heart and Vascular Centre Hamburg (UHZ) seeks to serve the holistic approach of an integrative ERAS protocol and to contribute to the refinement of ERAS in CS. In Addition, unlike many other ERAS programs that are designed to safely accelerate recovery for elderly and vulnerable patients in particular, this protocol is focused on patients with low risk profiles and stable clinical conditions, in order to retain ICU capacities for complex surgeries and critically ill patients. ERAS at UHZ started in February 2018 in selected patients undergoing minimally invasive heart valve surgery. We hypothesized, that the protocol leads to a reduction of hospital LOS without compromising patient safety and without adversely affecting the clinical results.

## Methods

### Study design and ethical approval

In this retrospective single-center cohort analysis performed at the University Heart and Vascular Center Hamburg, Germany, data of 101 consecutive patients undergoing elective minimally invasive heart valve surgery led by ERAS protocol and 111 patients receiving routine care was analyzed. It is in full accordance with the Declaration of Helsinki, released 2008 and was approved by our local Ethics Committee, Ethikkommission Hamburg, PV7050. There was no patients or public involvement in the development of this study. All patients included in this study gave their verbal consent for anonymized retrospective data analysis during the admission interview. For this retrospective study, the Ethics Committee did not require written informed consent. However, every patient admitted to the UHZ, is systematically asked for their consent to use their anonymized health- and treatment-related data in ongoing research projects [18].

From February 2018 to September 2020, 101 eligible patients matching the inclusion criteria were screened from the pool of all scheduled minimally invasive heart valve surgeries. They were contacted by phone and asked to attend a preoperative consultation 2–3 weeks prior to their surgery. Inclusion criteria was minimally invasive aortic valve or mitral valve surgery as well as age <75 years, sufficient physical condition and willingness to participate in the ERAS program, including preoperative preconditioning.

Mitral valve surgery included non-rib-spreading fully endoscopic 3D (Aesculap Einstein Vision, Tuttlingen, Germany) mitral valve repair (MVR) or replacement with or without concomitant tricuspid valve repair, left atrial ablation and left atrial appendage closure [19]. Procedures on the aortic valve included reconstructive techniques, such as bicuspid aortic valve repair and David- or Yacoub procedure [20], as well as aortic valve replacement including simultaneous supracoronary ascending aorta replacement or Bentall procedure [21]. Cardiac tumors, such as fibroelastomas or myxoma, that were accessible to minimally invasive surgery were also included.

Exclusion criteria was age >75 years, the need for complete median sternotomy (i.e. due to prior chest radiation, concomitant coronary artery disease requiring bypass surgery or re-operations after prior heart surgery), unwillingness to participate in ERAS program and severe comorbidities or conditions that increase the risk for peri- or postoperative complications or made them unsuitable for ERAS protocol. Furthermore, preexisting risk scores and prediction models were utilized, that identified prior heart surgery, extracardiac arteriopathy, obesity, elevated serum creatinine >150 μmol/L, nonelective and complex surgery as independent risk factors for failure of enhanced recovery [22, 23].

The control group contained 111 patients with the same inclusion and exclusion criteria, who either had surgery in a previous time period before the implementation of the ERAS protocol or did not want to participate.

The three hierarchically ordered primary endpoints are postoperative hospital length of stay (LOS), postoperative complications (beside nosocomial infections) and ICU LOS.

The secondary endpoints are duration of the heart lung machine, aortic cross clamp time (XCT), number of transfusions, nosocomial infections, delirium appearance of postoperative atrial fibrillation, re-operation and readmissions.

### ERAS program

This ERAS program was developed according to the Guidelines for Perioperative Care in Cardiac Surgery Enhanced Recovery After Surgery Society Recommendations as previously described in detail from our group [15, 18].

Core elements of contained a dedicated prehabilitation program with representatives of every involved profession one to three weeks before surgery. Present physical condition and frailty was assessed and patients were asked to perform daily exercising activities to sustain or improve physical capacity. A detailed patient education did not only focus on the forensic aspects of possible complications but served as psychologic preparation to improve mental readiness for surgery.

An intensified physiotherapy regimen started three hours after surgery and contained at least two daily units.

As limited rehab capacities might delay a timely discharge, rehab slots were organized during the first interview two weeks before surgery. Thus, a direct transfer to rehab was routinely performed in the majority of the patients unless wished otherwise.

### Patient characteristics

A detailed overview of demographic and clinical baseline characteristics of all enrolled patients (n = 212, 73% men, 54±11 years) is displayed in Table 1. Patients were predominantly men (73.3% in ERAS vs. 71.2% in control-group, p = 0.734) without relevant differences in disease characteristics between the two groups. Perioperative risk was low in both groups, with a tendency to a lower risk score in ERAS group (EuroSCORE II 0.67 (0.28) vs. 0.73 (0.38) in control group, p = 0.06). The ERAS group tends to be younger compared to the control group (56 (17) vs. 57.5 (13) years, p = 0.015). The primary analysis is adjusted for age and EuroSCORE II due to differences between treatment groups. There was no missing data for variables of interest.

**Table 1:** Preoperative baseline characteristic.

Baseline characteristic	ERAS group	Control group	p-value
	n = 101	n = 111	
Age (years)	56 (17)	57.5 (13)	0.015
Gender			
• male, n (%)	74 (73.3%)	79 (71.2%)	0.734
• female, n (%)	27 (26.7%)	32 (28.8%)	
LV-function, %	58±8	59±8	0.501
BMI	25.7±3.4	26.2±3.3	0.271
Creatinine (mg/dl)	0.9 (0.2)	0.94 (0.2)	0.166
Atrial fibrillation, n (%)	12 (11.9%)	16 (14.4%)	0.586
Diabetes mellitus Type II, n (%)	2 (2.0%)	7 (6.3%)	0.119
Hyperlipidemia, n (%)	20 (19.8%)	24 (21.6%)	0.744
Hypertension, n (%)	46 (45.5%)	60 (54.1%)	0.216
Coronary artery disease, n (%)	5 (5.0%)	11 (9.9%)	0.172
Chronic lung disease, n (%)	9 (8.9%)	13 (11.7%)	0.504
Prior stroke, n (%)	1 (1.0%)	3 (2.7%)	0.360
EuroSCORE II (%)	0.67 (0.28)	0.73 (0.38)	0.06

Values are presented as numbers (%), as mean ±SD or as median (IQR). P-values are considered descriptively.

### Statistical analysis

Statistical analysis was performed in collaboration with the Institute of Medical Biometry and Epidemiology (IMBE) of the University Medical Center Hamburg Eppendorf using SPSS Version 27.0 (IBM Corp, New York, USA). Categorical variables are given as percentages and absolute or relative numbers. Continuous variables are presented as mean ± standard deviation or median (IQR). If histograms showed a curve of normal distribution for continuous variables, data was compared using the unpaired two-sided t-test. Data without normal distribution was analyzed using Mann-Whitney U-Test. Categorical variables were analyzed using the chi-square test or the Fisher’s exact test, as appropriate. All p-values regarding the baseline variables are considered descriptively. The three hierarchical primary endpoints were analyzed using a multivariable linear regression or a logistic regression with adjustment for the clinically relevant baseline variables (age, aortic cross-clamp time (XCT)). If the previous endpoint is significant between the treatment groups, the following endpoint is evaluated confirmatory. Given the hierarchy of the endpoints, an adjustment of the significance level is not necessary. A p-value of <0.05 was considered statistically significant. The secondary endpoints were analyzed descriptively as mentioned above.

## Results

### Procedural characteristics

Receiving mitral valve or tricuspid valve surgery, atrial septal defect (ASD) closure, cryoablation or left atrial appendage (LAA) occlusion, 48.5% of patients in ERAS-group underwent right lateral mini-thoracotomy vs. 55.9% of patients in control group. 51.5% of patients in ERAS group had partial upper sternotomy vs. 44.1% in control group (p = 0.285), receiving aortic valve repair/replacement, aortic root or ascending aortic replacement. There is a relevant difference aortic cross clamp time between the treatment groups (77±28min vs. 107±41min, p<0.01). Therefore, the primary analysis is adjusted accordingly. An overview of performed procedures and intraoperative characteristics is shown in Table 2.

**Table 2:** Intraoperative and postoperative management.

Surgical variables	ERAS group	Control group	p-value
	n = 101	n = 111	
Mitral valve surgery	49 (48.5%)	62 (55.9%)	0.285
• mitral valve replacement	0	3 (2.7%)	
• mitral valve repair	48 (47.5%)	59 (53.2%)	
Aortic valve surgery	51 (51.5%)	49 (44.1%)	0.285
• aortic valve replacement	22 (21.8%)	26 (23.4%)	
• aortic valve repair	26 (25.7%)	20 (18%)	0.173
• concomitant aortic root surgery	4 (4%)	7 (6.3%)	
• ascending aorta replacement	1 (1%)	0	
• Bentall procedure	0	2 (1.8%)	
• fibroelastoma removal	4 (4%)	0	
CPB time (min)	130.5 (61)	147 (81)	0.076
XCT (min)	77±28	107±41	<0.01

Values are presented as numbers (%) or as mean ±SD. P-values are considered descriptively.

### Primary endpoints

The first primary endpoint (postoperative LOS) was statistically significant (p<0.01). Therefore, the second primary endpoint (postoperative complications) was confirmatory evaluated without showing a statistically significance. Hence, the last primary endpoint (ICU LOS) could only be evaluated descriptively. Here, a relevant difference between the treatment groups was shown.

### Clinical outcome

No intra- or perioperative complications were associated with ERAS-protocol. There was no in-hospital or 30-day-mortality in both groups. Transfusion was necessary in 11.9% of patients in ERAS group vs. 18.9% in control group (p = 0.158). There was no relevant difference in the occurrence of nosocomial infections (12.9% vs. 15.3%, p = 0.61), or other postoperative complications, which are summarized in detail in Table 3 (13.9% vs. 18%, p = 0.41). Reintubation was necessary in 3 patients that underwent ERAS-protocol vs. 4 patients in control group (p = 0.797). 24.8% of patients in ERAS group developed postoperative atrial fibrillation (poAF) compared to 15.3% in control group (p = 0.09). There was no difference in the number of readmissions to ICU (4% vs. 2.7%, p = 0.609).

**Table 3:** Postoperative complications.

Postoperative data	ERAS group	Control group	p-value
	n = 101	n = 111	
Hospital LOS, days	7 (3)	8 (4)	<0.01
ICU LOS, hours	18.5 (6)	26.5 (29)	<0.01
Postoperative LOS, days	6 (2)	7 (1)	<0.01
ICU readmission, n (%)	4 (4%)	3 (2.7%)	0.609
Reintubation necessary, n (%)	3 (3%)	4 (3.6%)	0.797
Transfusion necessary, n (%)	12 (11.9%)	21 (18.9%)	0.158
Redo surgery	7 (6.9%)	9 (8.1%)	0.746
• Valve related	4 (4%)	1 (0.9%)	0.143
• Bleeding	3 (3%)	8 (7.2%)	0.165
• Pericardial tamponade	0	2 (1.8%)	0.175
Pacemaker, n (%)	3 (3%)	4 (3.6%)	0.797
Nosocomial Infections, n (%)	13 (12.9%)	17 (15.3%)	0.61
• Pneumonia	3 (3%)	9 (8.1%)	0.106
Postoperative LV-function, %	52±8	52±9	0.65
Postoperative complications, n (%)	14 (13.9%)	20 (18%)	0.41
• Delirium, n (%)	5 (5%)	7 (6.3%)	0.67
• AV-Block, n (%)	4 (4%)	6 (5.4%)	0.62
• Acute coronary syndrome, n (%)	2 (2.0%)	1 (0.9%)	0.506
• Stroke, n (%)	2 (2.0%)	2 (1.8%)	0.924
• poAF, n (%)	25 (24.8%)	17 (15.3%)	0.085
Readmission from rehab, n (%)	16 (15.8%)	11 (9.9%)	0.196
• Pleura effusion	4 (4%)	3 (2.7%)	
• Pericardial effusion	5 (5%)	2 (1.8%)	
• Dyspnoe	1 (1%)	0	
• Valve dysfunction	1 (1%)	0	
• Organic psychosyndrome	2 (2%)	0	
• Atrial fibrillation	2 (2%)	1 (0.9%)	
• Wound infection	0	1 (0.9%)	
• Other Reasons	1 (1%)	4 (3.6%)	
• Intervention necessary	8 (7.9%)	4 (3.6%)	

Values are presented as numbers (%), as mean ±SD or as median (IQR). P-values are considered descriptively.

Surgical revision for bleeding, valve malfunctioning or pericardial tamponade that was necessary immediately or in the course of the hospital stay is summarized under redo surgery and was necessary in 8.9% in ERAS group vs. 11.7% in control group (p = 0.504). 4% in ERAS group were valve related redo surgeries vs. 0.9% in control group (p = 0.143). Surgical revision because of bleeding occurred in 3% in ERAS group vs. 7.2% in control group (p = 0.165). Permanent pacemaker implantations were also comparably similar (3% vs. 3.6%, p = 0.797).

There was no difference in the number of readmissions from rehab after 30 days (15.9% vs. 9.9%, p = 0.196). In ERAS group, a relevant decrease in hospital LOS (7 (3) days vs. 8 (4) days, p<0.01) as well as a significant decrease in postoperative LOS (6 (2) days vs. 7 (1) days, p<0.01) was shown. ICU LOS was significantly shorter (18.5 (6) hours vs. 26.5 (29) hours in control group, p<0.01).

## Discussion

### Patient selection and education

Since a small proportion of approximately 10–15% of patients develop 80–90% of complications, perioperative care is suggested to be further individualized [24]. In this program, a dedicated patient selection with exclusion of complex surgery and elderly patients >75 years, utilization of EuroSCOREII to predict in-hospital mortality, LOS and specific postoperative complications [25] reliably ensured hemodynamic stability after surgery and made way for uncomplicated immediate extubation in the OR.

The physiotherapeutic assessment three weeks prior to surgery is meant to detect and exclude frail patients and ascertain suitability for our demanding protocol. At the same time, it gives motivational benchmarks that patients can try to get back to in the course of postoperative convalescence. Furthermore, daily exercising activities before surgery demonstrated a decline in sympathetic over-reactivity and an improved insulin sensitivity [26, 27]. Additionally, being familiar with the execution of some exercises prior to surgery is suggested to help overcome postoperative phlegm and reluctance. A number of randomized controlled trials was able to demonstrate that such prehabilitation programs result in an improved physical and mental readiness for surgery, a reduction in ICU and hospital LOS and improved transition from hospital to the community [28–30].

Nevertheless, open heart surgery remains to be a demanding turning point in many patients lives. Even though some patients might feel noticeable restrictions in their everyday life caused by the disease, most of them will live a more or less independent and self-regulated life up to the point of hospitalization. Tubes, catheters and restrictions might limit mobility, regulations and schedules need to be followed, which marks a severe interference in a patient’s autonomy. In this ERAS protocol education and empowerment of patients exceeds the traditional form of information and consent, which is usually focused on the forensic aspects of possible complications. It is rather designed to obtain the patient as an active and relevant actor in their own process of healing, to improve mental and physical readiness for surgery and to help them regain their autonomy as soon as possible. Working off the imbalance of knowledge and creating an environment of shared decision making allows for reasonable expectations on the patient side and has shown to reduce fear and postoperative analgetic use [6, 31]. In turn, postoperative convalescence might be improved by a reduction of fear and analgetic use [32, 33].

### Immediate extubation and intensive care management

Safety of an early extubation and its positive impact on ICU LOS was demonstrated by numerous studies [34–36]. A decisive factor in the reduction of shorter ventilation times goes back to the establishment of short acting narcotics that allow for quicker extubation [36, 37]. Even an immediate extubation after off-pump coronary artery bypass surgery proved to be safe and feasible [38], although Montes et al. demonstrated that an extubation in the OR might be safe but has no effect on ICU or hospital LOS [39]. However, positive end-expiratory pressure during mechanical ventilation impedes RV ejection [40]. Considering every patient after heart surgery at risk for cardiac complications, we still identified minimalizing mechanical ventilation to be a core element of our ERAS protocol. Out of three patients who had to be reintubated, one patient had a stroke related seizure and two patients suffered from respiratory depression due to extensive pleural effusion days after surgery. Compared to four patients undergoing reintubation in the control group for similar reasons (two stroke related seizures and two respiratory depressions), we found the advantages to be preponderant, as we found significantly shorter lengths of stay and a numeric reduction of possibly ventilator-associated pneumonia in ERAS group (3% vs. 8.1%, p = 0.106). Intubation for surgical revisions, e.g. before rethoracotomy, was not included in the cases mentioned above. Furthermore, transferring already extubated patients to the ICU might prevent the staff from keeping patients asleep who are respiratory stable enough to be extubated because of a potentially stressful work environment and reduces nursing tasks.

Although immediate extubation was not utilized in the following case, Ender et al. demonstrated that for CS patients undergoing their unique fast-track concept, a direct transfer from the OR to a specifically opened postanesthetic care unit (PACU) without an intermediary stay on the ICU was feasible without compromising patient safety [35]. It is indeed worth mentioning, that 14% of these patients had to be transferred to ICU eventually. On the one hand, a reason might be a greater variety of complex surgery with patients undergoing multiple valve and combined procedures, and on the other hand there were limited opening hours on the PACU in Leipzig, necessitating an admission to ICU if patients were not hemodynamically stabile enough to be transferred to an intermediate care ward on 6:30pm on the day of surgery.

At UHZ, a careful patient selection and the new establishment of an overnight 24 hours PACU should allow for a safe patient transfer directly to the low care ward, entirely skipping ICU or intermediate care ward.

It cannot be ignored, that exclusion of elderly or high risk patients is an unconventional approach for ERAS protocols, that are typically focused on vulnerable patients in particular. However, we want to shine light on the growing importance of limited ICU resources, which is why it is designed to let low risk patients skip ICU entirely and thus retain ICU capacities for the critically ill. Due to the relatively new establishment of the PACU24, resources for overnight care are still in development. Therefore, most patients included in this study were directly transferred to ICU. Conducted cases, however, provide an indication that skipping ICU entirely will potentially find its way into this ERAS protocol.

### Aortic cross clamp time

Aortic cross clamp time (XCT) is an independent predictor of mortality, morbidity and prolonged hospital LOS in CS patients [41, 42]. In this study, a relevant difference in XCT between patients undergoing ERAS protocol and control group was shown and is in need of explanation.

Patients with low risk profiles undergoing isolated valve surgery may often be operated by aspiring young surgeons. After implementation of our ERAS protocol, there was much importance attached to the procedures being performed by highly trained surgeons with great expertise to guarantee the best possible outcome. This is why XCT in control group probably better reflects real-world experience in a university teaching hospital with many different levels of skills.

However, in this study univariate regression models showed a minimal effect on hospital LOS and no relevant effect on ICU LOS or any major postoperative complication to be significantly associated with XCT.

### Clinical implications

The success story of ERAS in a number of surgical disciplines made comprehensive protocols in CS gradually emerge, that, along with a reduction of hospital LOS, also indicated potential medical benefits. Results from Williams et al., who contributed to ERAS in CS with a retrospective comparison of 443 patients undergoing ERAS protocol and 489 historic patients in routine care, showed a reduction of gastrointestinal complications and increased staff and patient satisfaction [43], while a randomized trial of Li et al. could demonstrate a reduction of major postoperative complications such as acute renal failure, stroke or heart block [44]. A potential economic benefit of up to 1900€ per patient was demonstrated during the pilot phase of this study at the UHZ [45]. Taking these findings into account, our confidence is strengthened that this unique protocol will hopefully lead to improved clinical outcomes and contribute to an extension of ERAS programs in CS.

At the same time, decreasing lengths on CPB and emergence of less invasive techniques might furthermore increase the number of patients being eligible for enhanced recovery after CS. Sutureless aortic valve protheses offer an alternative for multiple valve or high risk surgery to reduce CPB time [46]. However, higher rates of paravalvular leaks and permanent pacemaker implantations question possible advantages over stented protheses or transcatheter valves and do not suggest a routine use in low risk patients at the moment [47]. On the other hand, encouraging early data from a novel beating heart mitral valve repair system indicates a proceeding development of “interventional” CS, that might be contributing to quicker convalescence and greater ICU capacities [48].

Even though Engelmann et al. elaborated all the different aspects of ERAS in CS and presented an inviting piece for educational and planning purposes, it soon becomes clear, that implementation of such a protocol is a complex and demanding task that requires permanent self-evaluation and the willingness to break with long-established practices [49]. To address this, a weekly feedback round with representatives of every involved profession and daily ward rounds for all included patients with subsequent case discussions was organized. As recommended by Salenger et al., who published a guideline for successful implementation of ERAS [49], it is intended to facilitate the way for necessary changes into clinical practice.

In surgical disciplines in particular, a traditionally conservative culture of holding on to reliable habits is understandable. Nevertheless, the future of enhanced recovery and its feasibility is based on clinicians who can ignite enthusiasm over a permanent vision of how to provide better care. New measures of care are necessary, that do not only strive towards satisfying surgical results and an early discharge, but towards a patient-centered individualization of health care.

## Limitations

Validity of data in a retrospective study design is limited by nature. Instead of randomization, patient selection was performed and differences in baseline variables occurred, which were adjusted using a multivariate regression model. However, results of the regression model demonstrated minimal influence of addressed variables for all of the primary and secondary endpoints. Indeed, the data collected in this study made way for the INCREASE-study (Interdisciplinary Perioperative Care in Minimally-invasive Heart Valve Surgery, NCT04977362), which is a randomized clinical trial that started in June 2021 and is expected to provide high quality data about the organization and execution of our ERAS protocol in the minimally invasive treatment of heart valve pathologies and their potential transfer into standard-of-care treatment.

## Conclusion

The main finding of this study is that the ERAS protocol for minimally invasive heart valve surgery is safe and feasible in selected patients and an elective setting. Clinical outcomes demonstrated a non-inferiority compared to routine care while hospital LOS was significantly shortened and ICU LOS was relevantly reduced, indicating that this protocol might be transferred into standard of care treatment. If these effects persist in a randomized controlled trial, needs to be explored.

## Abbreviations

CPB: cardiopulmonary bypass
CS: cardiac surgery
ERAS: enhanced recovery after surgery
ICU: intensive care unit
LOS: length of stay
OR: operating room
poAF: postoperative atrial fibrillation
UHZ: University Heart and Vascular Center Hamburg
XCT: aortic cross clamp time
